# Prostatic Inflammation Induces Urinary Frequency in Adult Mice

**DOI:** 10.1371/journal.pone.0116827

**Published:** 2015-02-03

**Authors:** Sanghee Lee, Guang Yang, Wade Bushman

**Affiliations:** 1 Department of Urology, University of Wisconsin-Madison School of Medicine and Public Health, Madison, Wisconsin, United States of America; 2 Carbone Cancer Center, University of Wisconsin-Madison School of Medicine and Public Health, Madison, Wisconsin, United States of America; 3 Cellular and Molecular Biology Program, University of Wisconsin-Madison School of Medicine and Public Health, Madison, Wisconsin, United States of America; Northern Institute for Cancer Research, UNITED KINGDOM

## Abstract

Lower urinary tract symptoms (LUTS) including urinary frequency and nocturia are common in aging men. Recent studies have revealed a strong association of prostatic inflammation with LUTS. We developed an animal model of bacterial induced, isolated prostatic inflammation and examined the effect of prostatic inflammation on voiding behavior in adult C57BL/6J mice. Prostatic inflammation was induced by transurethral inoculation of uropathogenic *E. coli—1677*. Bacterial cystitis was prevented by continuous administration of nitrofurantoin. Hematoxylin and eosin (H&E) staining and bacterial culture were preformed to validate our animal model. Voiding behavior was examined by metabolic cage testing on post-instillation day 1 (PID 1), PID 4, PID 7 and PID 14 and both voiding frequency and volume per void were determined. Mice with prostatic inflammation showed significantly increased voiding frequency at PID 1, 7 and 14, and decreased volume per void at all time points, as compared to mice instilled with saline and receiving nitrofurantoin (NTF). Linked analysis of voiding frequency and voided volumes revealed an overwhelming preponderance of high frequency, low volume voiding in mice with prostatic inflammation. These observations suggest that prostatic inflammation may be causal for symptoms of urinary frequency and nocturia.

## Introduction

Lower urinary tract symptoms (LUTS) are common in aging men. They include urinary frequency, urgency, nocturia, weak urinary stream, straining to void, and a sense of incomplete emptying [1, 2]. These symptoms have historically been attributed to prostatic enlargement and an increase in outlet resistance with secondary effects on bladder function [3–5]. However, recent studies suggest the etiology and pathogenesis of LUTS to be multifactorial and more complex than a simple function of prostatic enlargement. In particular, the presence of acute and chronic inflammation in the prostate has been associated with LUTS [6–8]. In a prospective study of autopsy specimens, chronic inflammation was found (primarily in the transition zone) in 75% of prostates affected by BPH as compared to only 50% of prostates not affected by BPH [9]. Prostate biopsy of 8224 men enrolled in the REduction by DUtasteride of prostate Cancer Events (REDUCE) Trial revealed inflammation in over three quarters of the biopsies [7]. And, evidence of inflammation on baseline biopsy in the Medical Therapies of Prostate Symptoms (MTOPS) trial correlated with symptomatic progression, risk for urinary retention and need for surgery [7, 10]. The basis for association of inflammation with LUTS is a subject of considerable interest. It has been postulated that chronic inflammation may produce prostatic fibrosis and that loss of compliance impairs opening of the prostate and bladder neck during voiding—thus contributing to bladder outlet obstruction. It has also been postulated that prostatic inflammation might influence bladder sensation and function [11–23].

The etiology of prostatic inflammation is unknown. However, urine refluxes freely into the prostatic ducts and provides an obvious route for bacterial entry [24]. While frank bacterial prostatitis is relatively uncommon [25] bacteria were isolated from 38% of prostate specimens obtained surgically from patients with sterile urine preoperatively [26]. Further, a broad prevalence of colonization by non-culturable organisms has been suggested by PCR assays demonstrating bacterial 16S ribosomal RNA in prostate biopsies [27]. The potential significance of this finding is the apparent association of bacterial colonization with histologic evidence of inflammation [28].

## Materials and Methods

### Animals

This study was carried out in strict accordance with the recommendations in the Guide for the Care and Use of Laboratory Animals of the National Institutes of Health. The protocol was approved by the Institutional Animal Care and Use Committee of the University of Wisconsin-Madison (approval number: M02448). All procedures in this study were performed with 8~12-week old C57BL/6 male mice (Jackson lab, Bar Harbor, MA). All transurethral inoculation procedure was performed under isoflurane anesthesia and all sacrifice was performed under isoflurane anesthesia immediately followed by cervical dislocation. All efforts were made to ameliorate animal suffering.

Transurethral inoculation of uropathogenic *E.coli 1677* (2 x 10^6^ per ml, 200 µl per mouse) was performed as previously described [29]. Briefly, polyethylene tubing (I.D. .86mm O.D. 1.27mm, Clay Adams, New York, NY) is slipped over a 27G1/2 needle coated with Surgilube (Savage Laboratories, Nelville, NY) and inserted into the urethra of male mice to reach the prostatic urethra. Either 200ul of E. coli or PBS (control) are then injected with a 1ml syringe.

Nitrofurantoin (27.2ug/g)(Sigma-Aldrich, St. Louis, MO) was injected sc, b.i.d. beginning one day prior to transurethral instillation and continuing until the end of the experiment. Nitrofurantoin was prepared by dissolving 50 µg/µl of Nitofurantion in N, N-Dimethylformamide (Sigma-Aldrich, St. Louis, MO) and diluting in a solution of 10% (v/v) ethanol (Sigma-Aldrich, St. Louis, MO), 40% (v/v) PBS (Life Technologies, Carlsbad, CA), and 50% (v/v) PEG400 (Center Valley, PA) to a final concentration of 6.8 µg/µl.

### Histology

The anterior prostate (AP), dorsal lateral prostate (DLP) and ventral prostate (VP) lobes, bladder and seminal vesicle (SV) were harvested, rinsed in DPBS and cut saggitally. The left half was fixed in 10% formalin (Sigma-Aldrich, St. Louis, MO), embedded in paraffin and serially sectioned (5µm) for Hematoxylin (Thermo Scientific, Waltham, MA) & Eosin (Anatech LTD, Battle Creek, MI) staining. Inflammatory infiltrate and other histological changes were noted. N = 4 mice from each group (Naïve +NTF, Saline +NTF, E.coli + NTF and E.coli only) and for each time point (PID 2 and 14) were used (total n = 32).

### Bacterial count

The right half of the sagittal tissue section was dissected to isolate the AP, DLP, VP, bladder and SV. These tissues were weighed and then homogenized in DPBS and cultured on Levine Eosin Methylene Blue Agar plates (BD, Sparks, MD). Eosin Methylene Blue Agar plates have been utilized to selectively detect gram-negative bacterial as previously described [30]. Bacterial colonies on the plates were counted and a number of bacteria per mg of tissue calculated. N = 4 mice from each group (Naïve +NTF, Saline +NTF, E.coli + NTF and E.coli only) and for each time point (PID 2 and 14) were used (total n = 32).

### Metabolic Cage Tests

We used metabolic cages to analyze the micturition pattern of infected and control mice. Mice were placed individually in a metabolic cage and allowed to acclimate for one hour. Micturition was monitored over the next 4 hours by placing laminated paper (Advantec, Japan) under each metabolic cage and noting the number, volume and pattern of micturition. Laminated paper (Filter Paper Qualitative Advantec 240mm, Tokyo Roshi Kaisha, Ltd, Japan) was changed at 40 minutes, then 1 hour 20 minutes later providing collection times of 40 minutes, 1 hour 20 minutes and 2 hours, for a total of collection time of four hours with the three papers. These collection times were determined by preliminary observational studies as optimal to prevent overlap of void stains on the paper. Water was withheld beginning with the acclimation period and throughout the experiment. The collected papers were scanned and imaged under UV light by Foto/Analyst Investigator Eclipse (Fotodyne Incorporated, Hartland, WI) to visualize urine stain. Micturition patterns were analyzed using two different categories; voiding frequency and volume per void. Data were collected by a blinded observer.

This analysis was based on the Voided Stain On Paper (VSOP) method previously described [31]. Laminated paper is placed under the wire mesh bottom of each metabolic cage. Urine stains on the paper are analyzed to determine voiding behavior and voided volumes. We established three distinct standard curves for circle, oval and corner shaped stains using known volumes of mouse urine. Volumes of 20 µl, 40 µl, 80 µl and 160 µl (n = 3 per volume) mouse urine were spotted on control laminated paper in circle, oval and corner shapes to mimic general voiding patterns of mice. A total number of 36 stains were used to establish the three standard formulas. The area of voided stains in the metabolic cage tests was measured by Image J. The area was then converted into a volume based on one of the three different standard formulas for circle, oval or corner shaped void. (Circle shape: y = 6.457x^1.1166^, R^2^ = 0.99904, Oval shape: y = 8.0653x^1.0727^, R^2^ = 0.99992, Corner shape: 9.8004x^1.08^, R^2^ = 0.99987)

### Statistics

Comparison between *E.coli* + NTF and Saline + NTF mice were performed by the paired t-test. We employed ANOVA with multiple comparisons using Fisher’s protected least significant difference test. Prior to analysis, all values were rank-transformed in order to better meet the assumptions of ANOVA. Comparison percentiles of mice showing LVHF void between *E.coli* + NTF and Saline + NTF mice were performed by Fisher’s exact test. P-values less than 0.05 were considered as significant. All analysis was performed using SAS statistical software version 9.1 and 9.2 (SAS Institute Inc., Cary, NC).

## Results

### Development of an animal model of isolated prostatic and seminal vesicle inflammation

**Fig 1:**
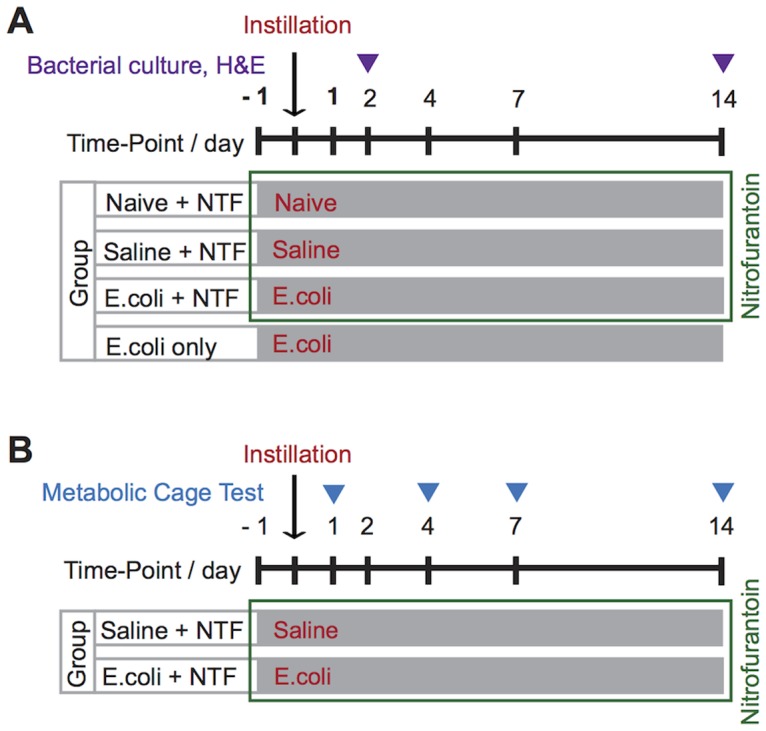
Experimental design to study the effects of isolated prostatic inflammation on voiding behavior. For mice receiving NTF, NTF was injected sc, twice daily, beginning one day prior to transurethral instillation and continuing until the end of the experiment. Either saline or uropathogenic *E.coli 1677* (2 x 10^6^ per ml), 200 µl per mouse, was instilled transurethral. (A) Histologic analysis and tissue culture was performed at the time points indicated for mice in the Naïve + NTF, Saline + NTF, *E.coli* + NTF and *E.coli* only groups. (B) Voiding behavior of mice in Saline + NTF and *E.coli* + NTF groups was determined by metabolic cage tests at PID 1, 4, 7 and 14.

**Fig 2:**
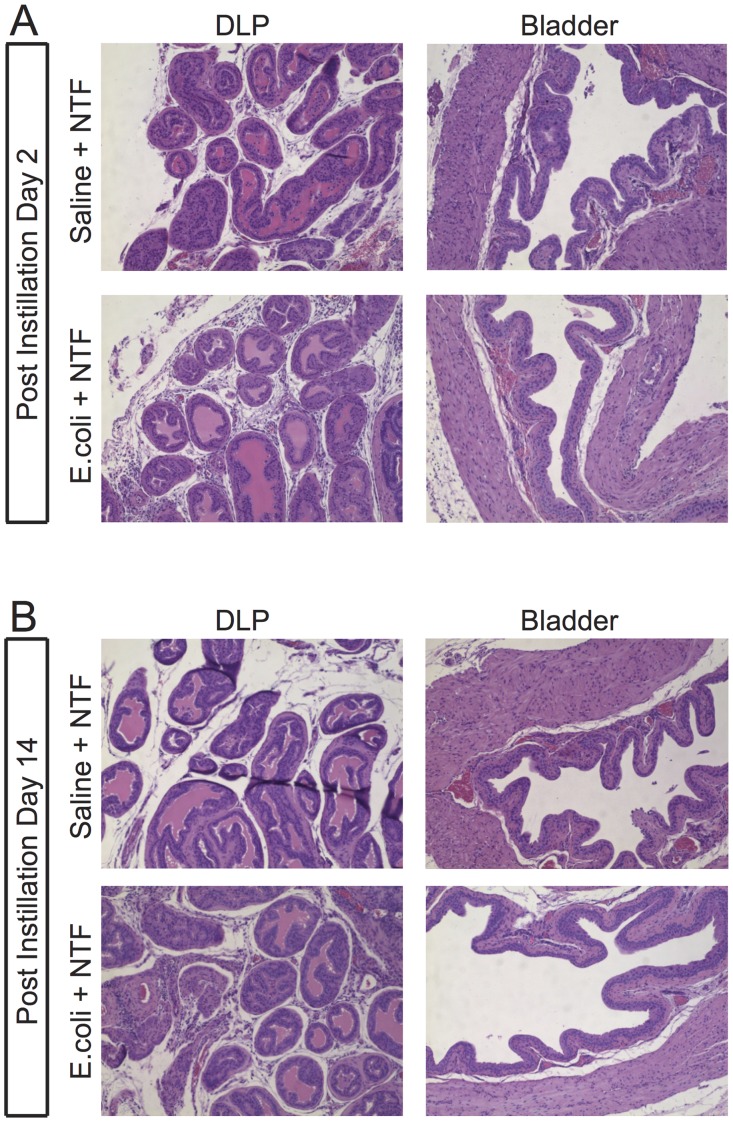
*E.coli 1677*-induced prostatic inflammation. Representative H&E stained sections of the DLP (A, C, E, G) and bladder (B, D, F, H) of mice in the Saline + NTF and *E.coli* + NTF groups at PID 2 and PID 14, respectively.

We previously developed a mouse model of prostatic inflammation induced by trans-urethral instillation of uropathogenic *E.coli 1677* into adult male mice [29, 32, 33]. Prostatic infection is generally accompanied by bacterial cystitis. To prevent bacterial cystitis mice were administered nitrofurantoin (NTF) daily beginning one day prior to bacterial instillation. NTF is concentrated in the urine but does not accumulate in tissues to a significant degree [34, 35]. The result is effective urinary prophylaxis that does not interfere with prostatic infection. Studies were performed to confirm that inoculation of E. coli with daily NTF administration produced prostatic inflammation without evidence of bacterial cystitis (Figs. 1, 2, Table 1).

**Table 1:** Bacterial titer of the bladder and prostate.

	**Post-instillation Day 2**		**Post-instillation Day 14**	
	**Bladder**	**Prostate**	**Bladder**	**Prostate**
Naïve + NTF	0	0	0	0
Saline + NTF	0	0	0	0
E.coli + NTF	8.81	55573.89	0	195.71
E.coli only	3317.26	61211.94	0	40.96

Number of colonies per mg of tissue of the bladder and prostate of mice in the Naïve + NTF, Saline + NTF, *E.coli* + NTF and *E.coli* only groups at PID 2 and 14 (total n = 32, n = 4 for each group at each time-point).

The prostate and bladder were harvested from mice at post-instillation day 2 (PID 2) and 14 (PID 14) and examined for evidence of inflammation and tissue injury. As expected, saline-instilled mice treated with NTF exhibited no inflammation in either prostate or bladder (Fig. 2). In mice instilled with *E.coli* and receiving NTF there was robust inflammation in all prostate lobes but no evidence of bladder inflammation. Seminal vesicle inflammation was present at PID 14 (data not shown). Mice instilled with *E.coli* without NTF exhibited diffuse epithelial injury and an intense inflammatory infiltrate in the bladder in addition to inflammation of the prostate lobes (data not shown). The contrast with absence of bladder inflammation in the *E.coli* + NTF treated animals is striking.

The efficacy of NTF prophylaxis was confirmed by bacterial culture (Table 1). Culture of bladder and prostate tissues from naive mice or saline-instilled mice treated with NTF revealed no evidence of infection. Culture of prostate tissue from mice inoculated with *E.coli* with or without NTF revealed robust infection in both at PID 2. Bacterial counts were diminished at PID 14, consistent with previous studies showing that bacterial counts peak at PID 5 and then decrease [29]. Bladders from *E.coli*-instilled mice without NTF exhibited a large number of colonies at PID 2 (3300 colonies/mg of tissue) consistent with bacterial cystitis shown in histology; the bacterial count at PID 14 was zero, suggesting a spontaneous resolution of the infection. Bladders from *E.coli*-instilled mice receiving NTF exhibited an extremely small number of colonies at PID 2 (9 colonies/mg of tissue) and none at PID 14. The presence of a small number of colonies in the bladder of E. coli + NTF treated mice is best explained that bacterial drainage from the prostatic ducts to the bladder during/after transurethral instillation procedure does not result in active infection of the bladder. Taken together, the histological analysis and determination of bacterial titer demonstrate that NTF effectively prevents bladder infection and inflammation.

### Voiding Behavior: Frequency and Volume per Void

**Fig 3:**
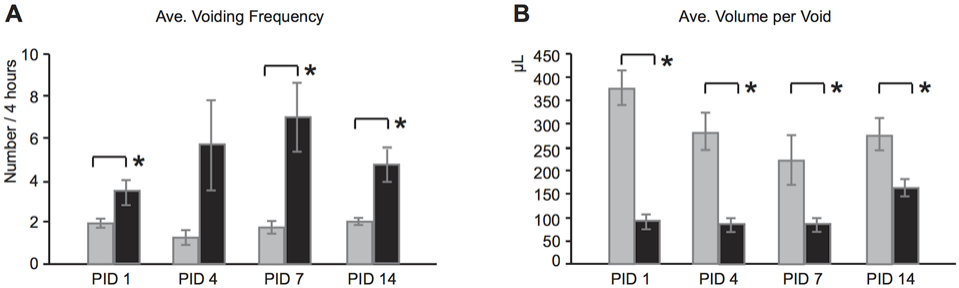
Voiding behavior of mice in the Saline + NTF and *E.coli* + NTF groups. (A) Voiding frequency (number) at PID 1, 4, 7 and 14. (B) Volume per void (μl) at PID 1, 4, 7 and 14 (B). n = 13 of Saline + NTF (gray bar), n = 12 of *E.coli* + NTF (black bar).

Voiding behavior was compared between mice instilled with either saline (n = 13) or *E.coli* (n = 12) and treated with NTF. Four time points over a 14 day period post-instillation were selected for comparison that correspond to initiation (PID 1), peak (PID 4) and resolving (PID 7, 14) stages of prostatic infection and inflammation [29]. Voiding frequency and volume per void were determined over a 4-hour period as described in the materials and methods. *E.coli*-instilled mice exhibited a significantly higher voiding frequency than controls at PID 1, 7 and 14 (Fig. 3, p < 0.05). Voiding frequency was also increased at PID 4 but the difference was not statistically significant. *E.coli*-instilled mice also exhibited a significantly reduced volume per void at all time points tested (Fig. 3, p < 0.05). Total voided volume was significantly decreased in *E.coli*-instilled mice on PID 1 and increased on PID 7 (data not shown). These fluctuations, likely resulting from physiologic fluid shifts associated with the acute and resolution phases of inflammation, do not explain the observed changes in voiding frequency and volume per void.

Given the general inverse relationship between voiding frequency and voided volume, the best analysis of effects on voiding behavior would take into account both parameters.

**Fig 4:**
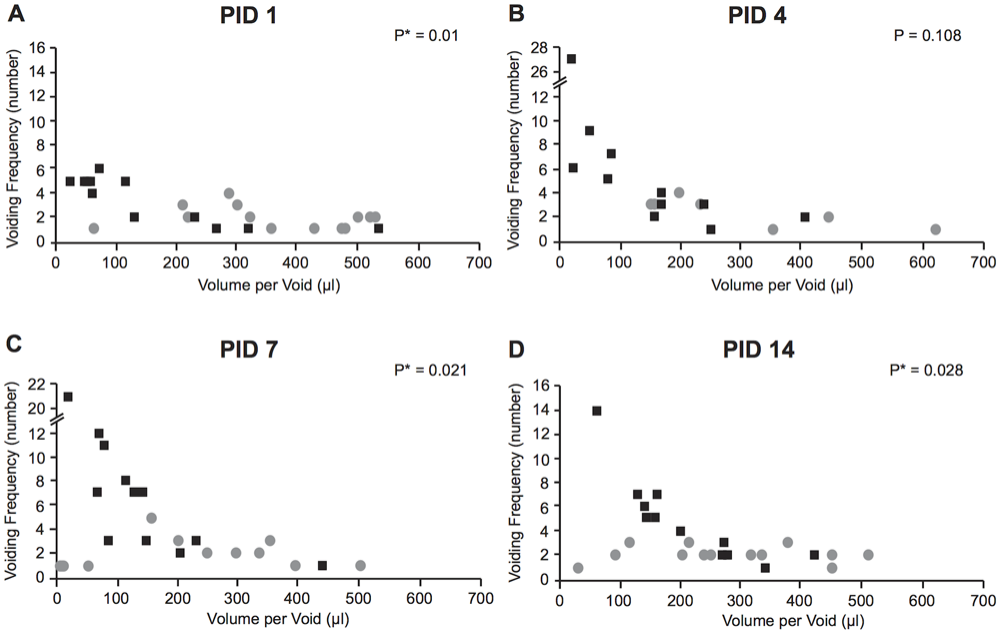
2D plot of voiding frequency and volume per void. Distribution of voiding frequency (number) and average volume per void (μl) for each mouse mice in the Saline + NTF (gray circle) and *E.coli* + NTF (black square) groups at (A) PID 1, (B) PID 4, (C) PID 7 and (D) PID 14. P-values were calculated by Multivariate Analysis (MANOVA). * statistically significant.

**Fig 5:**
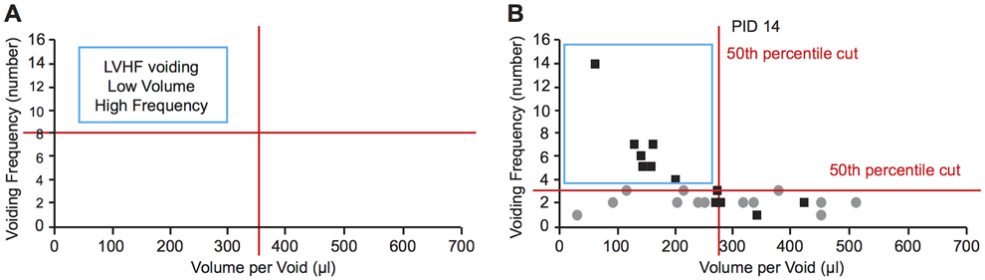
Combined analysis of voiding frequency and average voided volumes. (A) When average voided volume and voiding frequency are plotted together, the upper left hand corner is the domain of low frequency high volume (LVHF) voiding. The limits of this domain can be defined by percentile cut-points. (B) Plot of voiding frequency and average volume per void (μl) for each mouse mice in the Saline + NTF (gray circle) and *E.coli* + NTF (black square) groups at PID 14 with the LVHF domain defined by the 50^th^ percentile of frequency and volume.

When we plotted voiding frequency and volume per void for each mouse in each group at each time point on 2-D graph (Fig. 4), multivariate analysis (MANOVA) revealed significant differences at PID 1, 7 and 14. We predicted that prostatic inflammation would be associated with a preponderance of low volume and high frequency (LVHF) voiding (Fig. 5, A). To specifically examine this prediction, we established cut-off points for high voiding frequency and low average voided volume based on 90^th^, 75^th^, 50^th^, 25^th^ and 10^th^ percentiles of mice in the group of Naïve + NTF. The 90^th^, 75^th^, 50^th^, 25^th^ and 10^th^ percentiles for high urinary frequency were >5, >4, >3, >2, and >1. The 90^th^, 75^th^, 50^th^, 25^th^ and 10^th^ percentiles for low average volume per void were < 108.80, < 199.35, < 275.87, < 348.00 and >424.24 microliters, respectively. We then determined the percentage of *E.coli*-instilled mice satisfying the 90^th^ percentile for both high frequency and low volume. We then determined the percentage satisfying the 75^th^ percentile for both, and then the 50^th^, 25^th^ and 10^th^ percentiles for both. The same was done for the saline controls (Table 2). It is readily apparent from comparison of the 50^th^, 75^th^ and 90^th^ percentiles that LVHF voiding is found almost exclusively in the *E.coli*-instilled mice.

**Table 2:** LVHF voiding among mice in the Saline + NTF and *E.coli* + NTF groups.

		**Group**	**PID 1**	**PID 4**	**PID 7**	**PID 14**
Five Different Cut Points	10%	Saline + NTF	38.5	30.8	46.2	69.2
		E.coli + NTF	75	75 *	91.7 *	91.7
	
	25%	Saline + NTF	23.1	30.8	15.4	15.4
		E.coli + NTF	58.3	66.7	83.3 *	66.7 *
						
	50%	Saline + NTF	0	7.7	7.7	0
		E.coli + NTF	58.3 *	50 *	58.3 *	58.3 *
						
	75%	Saline + NTF	0	0	7.7	0
		E.coli + NTF	50 *	41.7 *	58.3 *	50 *
						
	90%	Saline + NTF	0	0	0	0
		E.coli + NTF	8.3	33.3 *	33.3 *	8.3

Table 2. Cut points were varied from the 10^th^ to 90^th^ percentile for both voiding frequency and average voided volume: 10^th^ percentile = 1 < voiding frequency, volume per void < 108.80; 25^th^ percentile = 2 < voiding frequency, volume per void < 199.35; 50^th^ percentile = 3 < voiding frequency, volume per void < 275.87; 75^th^ percentile = 4 < voiding frequency, volume per void < 348.00; 90^th^ percentile = 5 < voiding frequency, volume per void < 424.24). Note the preponderance of low volume and high frequency (LVHF) voiding among mice in the *E.coli* + NTF group at cut points at the 50^th^ percentile and above.

* statistically significant.

## Discussion

The purpose of this study was to test the hypothesis that prostatic inflammation could induce changes in voiding behavior. Prostatic inflammation was induced by transurethral instillation of adult male mice with uropathogenic *E. coli 1677*. To prevent cystitis, we administered the antibiotic NTF (Macrodantin,1-[[[5-nitro-2 furanyl]methylene]amino]- 2,4-imidazolidinedione), an agent that does not reach significant tissue levels but is highly concentrated in the urine [34, 35]. Controls included naïve animals treated with NTF and animal inoculated with saline vehicle and treated with NTF. Mice inoculated with *E. coli* and maintained on NTF exhibited persistent prostate infection and inflammation without evidence of either inflammation or infection of the bladder. Histologic examination of the seminal vesicle in each group (n = 4) at PID 2 showed no evidence of inflammation (data not shown). Examination at PID 14 showed seminal vesicle inflammation in both the *E. coli* and *E. coli* + NTF groups (n = 4). Metabolic cage testing 1, 4, 7 and 14 days after inoculation showed increased voiding frequency and decreased voided volumes in mice instilled with *E.coli* and receiving NTF. Since significant changes in voiding behavior occurred as early as PID 1, we attribute the changes to the effect of prostatic inflammation—recognizing that we cannot rule out a contributory effect of seminal vesicle inflammation at later time points. The effect of prostate inflammation was even more striking when we examined voiding frequency and voided volumes in tandem (Table 2). This revealed a clear-cut preponderance of LVHF voiding in this group.

Previous animal studies have shown an inverse relationship between voiding frequency and volume per void [36, 37], however, the two parameters are not perfectly correlated since the rate of urine production is variable. Voiding frequency will increase or decrease as a function of urine output if bladder capacity is unchanged, but bladder capacity and voided volumes may simultaneously be affected by the rate of bladder filling or changes in bladder sensation [38]. For this reason, we examined both voiding frequency and voided volumes in our analysis. When voiding frequency and voided volumes were plotted together (Fig. 4), we did not observe inverse relationship between voiding frequency and voided volume in the control group. This is most likely due to the relatively low average frequency of voiding during the 4-hour observation period. On the other hand, the inverse relationship was clearly evidence in the mice instilled with *E.coli* and treated with NTF. It is striking that over half these mice exceeded the 50^th^ percentile for high urinary frequency and low average voided volume at each time point. In contrast, less than 10% of the control mice exhibited this measure of volume/high frequency voiding. The advantage of analyzing voiding frequency and voided volume in tandem comes from negating the effect of variable urine output. The power of this approach is evidenced by the observation that analysis of frequency alone did not reveal significant differences at PID 4, whereas analysis of voiding frequency and voided volume in tandem revealed significant differences at multiple cut points.

Prostatic inflammation has been postulated to affect voiding by two different mechanisms—inducing fibrosis that impairs opening of the bladder neck and prostate during voiding and producing changes on bladder sensation and function by neural cross-talk [11–23]. We have shown that bacterial-induced inflammation does produce fibrosis of the mouse prostate but that significant fibrosis does not occur before 2 days post-inoculation [39]. Therefore, the change in voiding behavior observed here as early as one day post-inoculation cannot be attributed to fibrosis. There are three ways in which prostate inflammation could induce changes in bladder sensation and function. Release of inflammatory mediators such as prostaglandin, serotonin, ATP, histamine, bradykinin, and neurotropic factors such as NGF could exert collateral effects on bladder afferents [40–44]. Bladder behavior may be affected by neural crosstalk between the bladder and prostate. There is strong experimental evidence for neural cross-talk among the pelvic organs [11–21] and our laboratory has recently performed dual-label nerve tracing studies showing convergent of the prostate and bladder by neurons in the lumbosacral dorsal root ganglia (manuscript in preparation). Finally, hyper-sensitization of the pelvic afferents as a result of inflammation could increase sensitivity to bladder filling and changes in voiding behavior [45, 46]. It is quite likely that more than one and possibly all three mechanisms contribute to the observed increase in LVHF voiding associated with prostate inflammation.

Our model of bacterial-induced inflammation was developed with the support of the NIDDK as a tractable model to study the effects of inflammation on the prostate and lower urinary tract function. It utilizes inoculation with a uropathogenic strain of E. coli to produce infection and a robust inflammatory response. Both the acute and chronic inflammatory processes have been described, the leukocytic and inflammatory cytokine profiles characterized and the molecular signaling mechanisms involved in reactive epithelial hyperplasia identified [29, 33, 47]. Although acute and chronic inflammation in the human prostate cannot generally be attributed to a robust uropathogenic infection this model has proven valuable to provide proof of principle that both acute inflammation (1—and 4-days post-inoculation) and chronic inflammation (7—and 14-days post-inoculation) induce high frequency/low volume voiding. Further studies may be pursued using this model to determine if the induced changes are reversible with treatment and resolution of infection and inflammation or can be mitigated by administration of agents that blunt the inflammatory response or its neural-mediated effects.
